# Characterization of ApoJ-reconstituted high-density lipoprotein (rHDL) nanodisc for the potential treatment of cerebral β-amyloidosis

**DOI:** 10.1038/s41598-017-15215-w

**Published:** 2017-11-07

**Authors:** Sofía Fernández-de-Retana, Mary Cano-Sarabia, Paula Marazuela, Jose Luis Sánchez-Quesada, Annabel Garcia-Leon, Alex Montañola, Joan Montaner, Daniel Maspoch, Mar Hernández-Guillamon

**Affiliations:** 1 Neurovascular Research Laboratory, Vall d’Hebron Research Institute, Universitat Autònoma de Barcelona, Barcelona, Spain; 2 Catalan Institute of Nanoscience and Nanotechnology (ICN2), CSIC and the Barcelona Institute of Science and Technology, Campus UAB, Bellaterra, Barcelona, Spain; 3 Cardiovascular Biochemistry Group, Research Institute of the Hospital de Sant Pau (IIB Sant Pau), Barcelona, Spain; 4 Institució Catalana de Recerca i Estudis Avançats (ICREA), 08100 Barcelona, Spain

## Abstract

Cerebral β-amyloidosis is a major feature of Alzheimer’s disease (AD), characterized by the accumulation of β-amyloid protein (Aβ) in the brain. Several studies have implicated lipid/lipoprotein metabolism in the regulation of β-amyloidosis. In this regard, HDL (High Density Lipoprotein)-based therapies could ameliorate pathological features associated with AD. As apolipoprotein J (ApoJ) is a natural chaperone that interacts with Aβ, avoiding its aggregation and toxicity, in this study we propose to prepare reconstituted rHDL-rApoJ nanoparticles by assembling phospholipids with recombinant human ApoJ (rApoJ). Hence, rHDL particles were prepared using the cholate dialysis method and characterized by N-PAGE, dynamic light scattering, circular dichroism and electron transmission microscopy. The preparation of rHDL particles showed two-sized populations with discoidal shape. Functionally, rHDL-rApoJ maintained the ability to prevent the Aβ fibrillization and mediated a higher cholesterol efflux from cultured macrophages. Fluorescently-labelled rHDL-rApoJ nanoparticles were intravenously administrated in mice and their distribution over time was determined using an IVIS Xenogen® imager. It was confirmed that rHDL-rApoJ accumulated in the cranial region, especially in old transgenic mice presenting a high cerebral Aβ load. In conclusion, we have standardized a reproducible protocol to produce rHDL-rApoJ nanoparticles, which may be potentially considered as a therapeutic option for β-amyloid-related pathologies.

## Introduction

Cerebral β-amyloidosis is a major feature of Alzheimer’s disease (AD), characterized by the accumulation of β-amyloid protein (Aβ) in the brain. Aβ is originated by the sequential processing of the amyloid precursor protein (APP), primarily generating peptides constituted by 40- and 42-amino acids, Aβ_40_ and Aβ_42_, respectively. High concentrations of Aβ promote its aggregation into toxic species, such as oligomers and fibrils, within the brain extracellular space. In sporadic AD, in addition to the formation of neuritic plaques formed by the aggregation of Aβ species in the parenchyma, the intracellular accumulation of the hyperphosphorylated TAU protein induces progressive neuronal loss triggering cognitive and memory impairment^1^. Besides, 91% of sporadic AD cases also present vascular accumulation of Aβ, known as Cerebral Amyloid Angiopathy (CAA)^2^.

Several studies have implicated lipid/lipoprotein metabolism in the AD pathology. Firstly, the genotype of the lipid-carrier apolipoprotein E (ApoE) is the major genetic risk factor for developing sporadic AD^3^. The presence of the APOE ε4 allele is strongly associated with a greater incidence of AD, whereas the APOE ε2 allele is related with a lower risk of developing dementia^4^. In addition, diseases characterized by alterations in the lipid profile, such as diabetes mellitus type 2^5^, atherosclerosis^6^ and hypercholesteremia^7^ are also risk factors for developing AD^8^. These evidences, together with the association of cerebrovascular dysfunction in AD^9^, make the regulation of lipid metabolism a promising therapeutic approach to protect the AD-affected brain^10,11^. In this regard, HDL (High Density Lipoprotein)-based therapies have been also considered for the treatment of pathologies associated with Aβ deposition^8,12,13^. In the cerebral β-amyloidosis context, HDL levels (>55 mg/dL) in plasma were related with a lower risk of developing AD^6^. Furthermore, low plasma HDL-Cholesterol (HDL-C) was recently associated with higher Aβ binding, as measured using Pittsburgh compound-B positron emission tomography, in cognitively normal subjects and elderly subjects with mild cognitive impairment^14^.

The HDLs circulating in plasma are mainly comprised of apolipoproteinA-I (ApoA-I), cholesterol and phospholipids, and their principal role is to conduct the reverse cholesterol transport (RCT). However, plasma HDLs are extremely heterogeneous, presenting a diverse lipid and protein composition^15,16^, which may confer different functions associated with immunity regulation and vascular integrity^17^. In fact, multiple protective roles have been attributed to HDLs, such as anti-oxidant^18^, anti-apoptotic^19^, vasoprotective^20^ and anti-inflammatory properties^21^. The major apolipoprotein constituent of HDL in the brain is ApoE, followed by apolipoprotein J (ApoJ, also known as clusterin). ApoJ is a multifunctional heterodimeric protein which acts as a natural chaperone^22^. In AD brains, ApoJ is co-deposited with fibrillar Aβ in cerebrovascular and parenchymal lesions^23,24^. ApoJ also binds to Aβ in CSF and plasma^25,26^, and increased circulating levels of plasma ApoJ are associated with a higher prevalence and severity of AD^27,28^. On the other hand, the link between ApoJ and AD was highlighted in genome-wide association study that found a statistical association between a SNP within the CLU gene and the risk of suffering AD^29,30^. The rs11136000^C^ SNP in CLU gene was the associated with reduced ApoJ expression and increased risk of AD^31^, whereas the protective rs11136000^T^ was related with increased ApoJ expression in brain tissue and reduces the risk of AD^32,33^. These results propose the higher expression of rApoJ as a protective response against AD pathology. Moreover, experimental studies have shown that ApoJ is able to inhibit the aggregation of soluble Aβ^23^ and participates in the clearance of Aβ across the Blood Brain Barrier (BBB)^34–36^.

In this study, we have designed and prepared new functional nanoparticles to be considered as a novel therapeutic approach to ameliorate the features associated with AD. Because of the capacity of ApoJ to act as an Aβ chaperone, we have produced reconstituted HDL nanodiscs formulated with recombinant ApoJ (rHDL-rApoJ nanodiscs). The nanodiscs have been characterized in terms of size, morphology, Aβ binding and RCT *in vitro*. We have also determined the distribution of rHDL-rApoJ nanodiscs *in vivo* after a single intravenous (IV) administration using a transgenic mouse model of cerebral β-amyloidosis.

## Results

### rApoJ protein production and characterization

**Figure 1:**
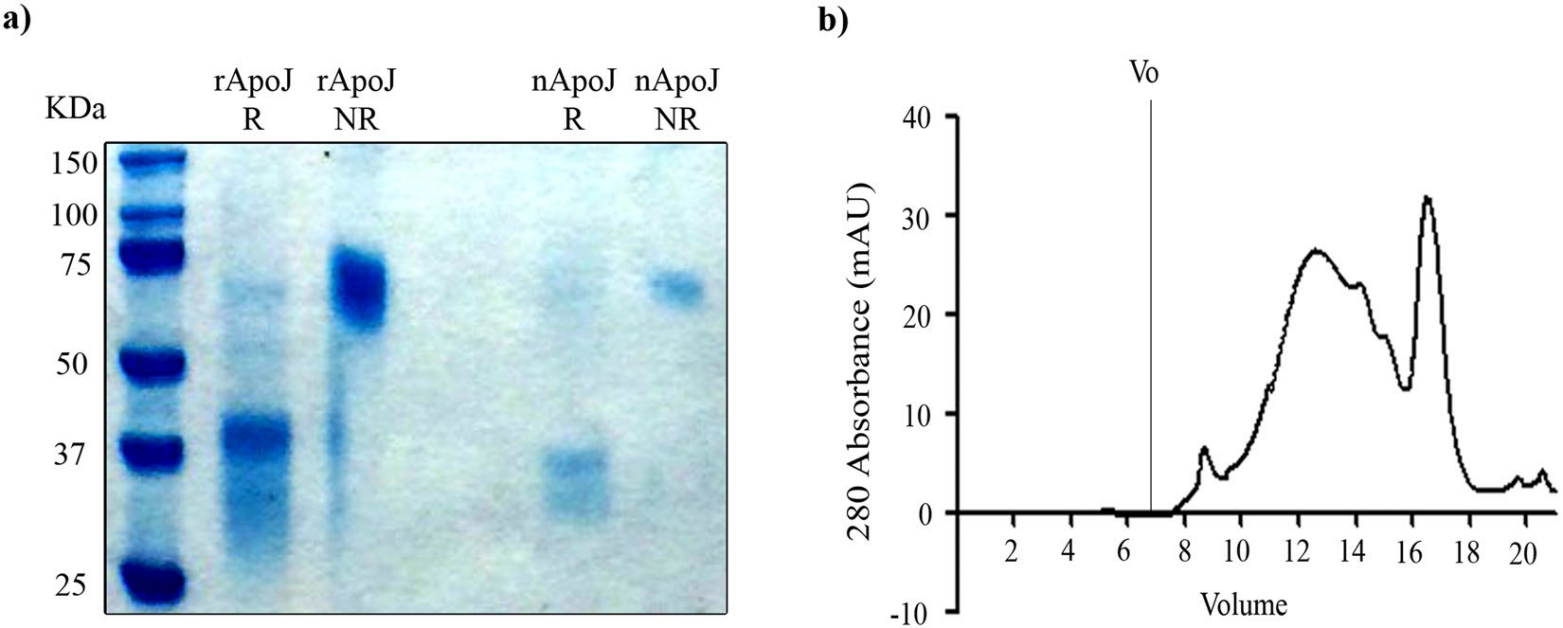
rApoJ characterization (**a**) SDS-PAGE of purified recombinant ApoJ (rApoJ) and native ApoJ purified from plasma (nApoJ) under both reducing (R) and non-reducing (NR) conditions. **(b)** SEC analysis of rApoJ. V_0_  = Void volume.

Highly pure secreted His-tagged recombinant ApoJ (rApoJ) protein was obtained through a single step purification from human cultured cell supernatants. The electrophoretic profile of rApoJ was compared with the native ApoJ purified from human plasma through Sodium dodecyl sulphate polyacrylamide gel electrophoresis (SDS-PAGE). First, under reducing conditions, the cleavage of rApoJ into α- and β-chains during its maturation was conserved, as two chains of ≈40 KDa were obtained. In contrast, the non-reducing conditions confirmed the maintenance of disulfide bonds and the heterodimeric state of rApoJ (≈77 KDa). On the other hand, the rApoJ showed a higher molecular weight due to the His-tag and the C-Myc epitope, which conferred a 3.30 KDa increase compared to the nApoJ (Fig. 1a). To test the aggregation proclivity of rApoJ, the purified recombinant protein was submitted to Size Exclusion Chromatography (SEC) analysis. Our results confirmed that rApoJ was prone to aggregation and the formation of heterogeneous high molecular weight oligomers as shown by the wide peak observed between 12–16 ml of the elution volume. Moreover, a homogeneous peak appeared at 16–18 ml of the elution volume with a maximum at 16.5 ml, corresponding to lesser aggregation state oligomers (Fig. 1b).

### rHDL-rApoJ purification in KBr density gradient ultracentrifugation

**Figure 2:**
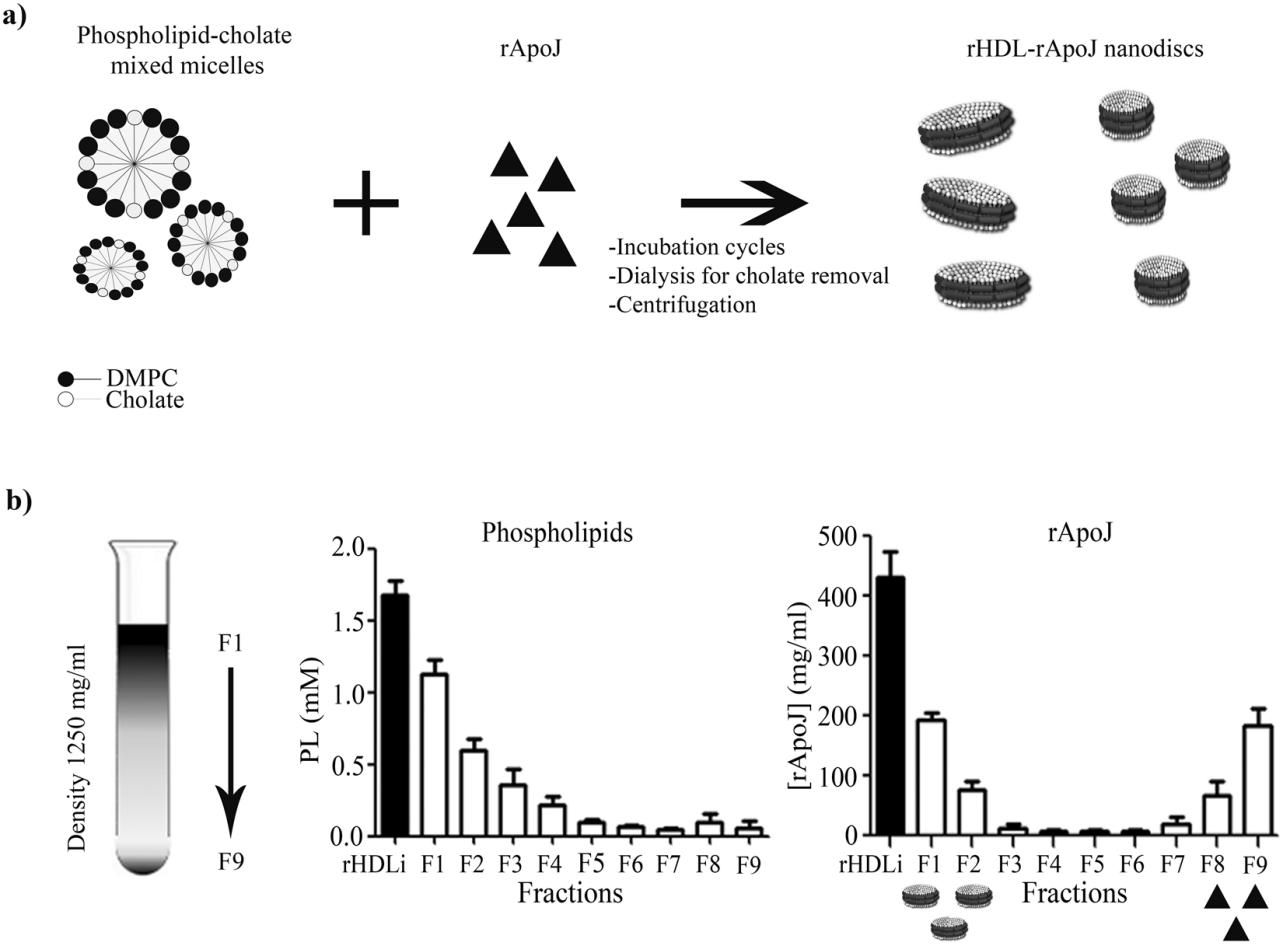
rHDL-rApoJ synthesis and purification. (**a)** Schematic representation of rHDL-rApoJ nanodisc synthesis. **(b)** Schematic representation of the purification process by KBr density ultracentrifugation. Phospholipid and rApoJ (protein) quantification in the different fractions (F1-F9) after the purification process. PL = Phospholipids. N = 6 independent experiments.

The preparation and purification protocols were optimized to maximize the rHDL-rApoJ nanodisc collection and resulted to be highly reproducible (Fig. 2a,b). The lower density of rHDL-rApoJ nanodiscs (<1250 mg/ml) in comparison to free rApoJ (1300 mg/ml) allowed the correct purification of nanodiscs through KBr gradient density ultracentrifugation. After the ultracentrifugation step in the KBr gradient, phospholipids and total protein amounts were measured in all fractions obtained (Fig. 2b). We confirmed that the concentration of lipids decreased from top-F1 to bottom-F9 fractions due to their low density. In contrast, the highest concentrations of rApoJ were found in the fractions F1-F2 and F8-F9. Because of the density switch caused by the lipidation of rApoJ, rHDL-rApoJ nanodiscs were present in F1 and F2 (d < 1250 mg/ml), whereas free rApoJ (unbound to the lipids, d > 1300 mg/ml) was mainly found in F8 and F9.

### rHDL-rApoJ nanoparticles characterization

**Figure 3:**
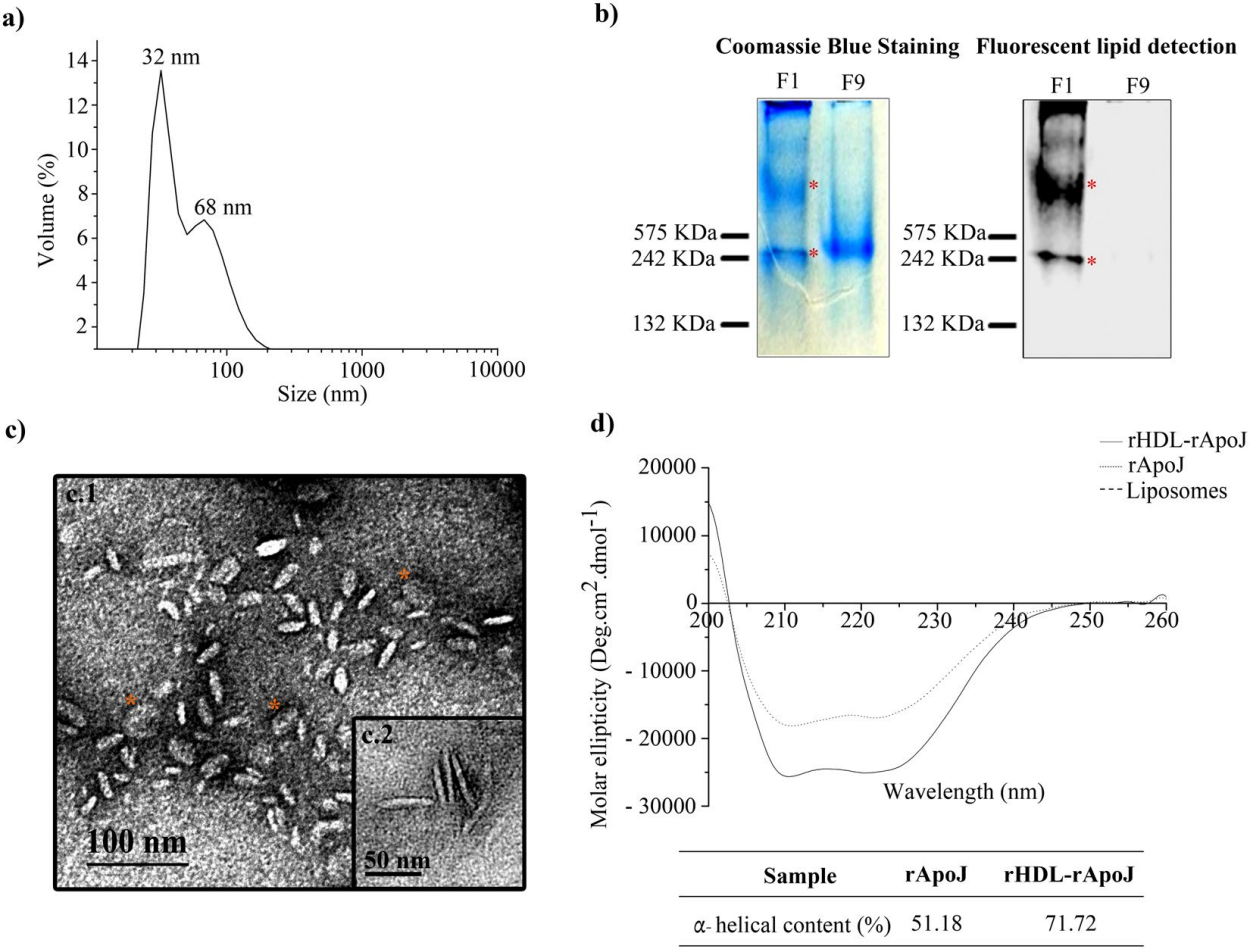
rHDL-rApoJ characterization. (**a**) Particle-size analysis by DLS showing the particle size after purification. **(b)** Native-PAGE analysis: Left panel; Coomassie Blue Staining, and, right panel; phospholipid fluorescence detection using the ODYSSEY imager. F1 refers to the first fraction of the purification step and corresponds to the purified rHDL-rApoJ nanodiscs. F9 refers to the ninth and last fraction of the purification step and corresponds to the discarded non-lipidated rApoJ. **(c)** Representative images of TEM showing two size populations of rHDL-rApoJ (F1) with a diameter of 24 ± 5 nm (C1) and 48 ± 2 nm (C2) both with 5 ± 1 nm of height. Orange stars indicate the face-on view of rHDL-rApoJ nanodiscs. **(d)** Circular Dichroism plot showing the non-lipidated rApoJ (dotted line), rHDL-rApoJ nanodisc (solid line) and the liposome (dashed line) spectra and the corresponding α-helical content calculation (%). Liposome ellipticity resulted 0 coinciding with the x-axis.

Purified rHDL-rApoJ nanoparticles were characterized by Dynamic Light Scattering (DLS), Native-PAGE (N-PAGE), Transmission Electron Microscopy (TEM) and Circular dichroism (CD). The methodology used for the rHDL-rApoJ preparation resulted highly reproducible, obtaining two homogeneous populations of rHDL-rApoJ nanoparticles after the purification, one centered at 30 ± 3 nm and the other at 70 ± 1 nm, according to the DLS analysis (Fig. 3a). Next, F1 (purified rHDL nanoparticles) and F9 (discarded free rApoJ) fractions obtained after the KBr density ultracentrifugation were analyzed by N-PAGE, and the protein content in the solution was determined by Coomassie blue staining, whereas the fluorescent lipids were detected using ODYSSEY Imager recoding (Fig. 3b). Our results confirmed that F1 contained rHDL-rApoJ particles, as shown by the co-detection of protein and fluorescent lipids from the same species. In fact, two different populations of rHDL-rApoJ particles appeared after the purification, as previously shown in the DLS analysis. In contrast to F1, when the densest fraction of the purification step (F9) was analyzed, only free protein was detected by Coomassie Blue staining whereas no lipids were detected, confirming the correct purification of rHDL-rApoJ nanodiscs.

The morphology and size of rHDL-rApoJ nanoparticles was determined by TEM. From the measurement of 1000 nanoparticles, we determined that the F1 fraction was constituted by two size populations of discoidal shaped particles, whose diameter means are centered on 24 ± 5 nm and 48 ± 2 nm. The nanodiscs thickness was 5 ± 1 nm in height (Fig. 3c; Supplementary Fig. S1). Most of nanodiscs are viewed edge-on, whereas others are viewed face-on in TEM (orange stars in Fig. 3c), confirming the discoidal shape of nanodiscs. The presence of two populations is in agreement with the results observed by DLS and N-PAGE (Fig. 3a,b). Moreover, the conformational changes that occurred in rApoJ due to its lipidation were studied by CD (Fig. 3d), where rHDL-rApoJ nanodiscs were compared to free rApoJ and liposomes prepared with the same phospholipid composition. As expected, the liposomes did not show any polarized-light absorption. Comparing free rApoJ with rHDL-rApoJ nanodiscs (rHDL-rApoJ), a gain in α-helical content was detected as result of the lipidation. The α-helical content calculated for free rApoJ was 51.18%, whereas this percentage increased up to 71.72% in the rHDL-rApoJ preparation, which demonstrated the structural ability of rApoJ to form rHDL particles and the underlying conformational change. Taking these results together, we could confirm the correct and reproducible preparation of rHDL-rApoJ nanoparticles, obtaining discoidal shape nanodiscs with 30 and 70 nm of diameter and 5 nm of height, generating an increase in the α-helical content of rApoJ. Resulting rHDL-rApoJ nanodiscs were stable after storage at −80 °C up to 3 months and after freeze-thaw cycles and did not show any difference in DLS diameter nor in TEM analysis after the dispersion of nanodiscs in different working buffers (TBS, PBS, KBr density solution).

### Study of rHDL-rApoJ functionality

**Figure 4:**
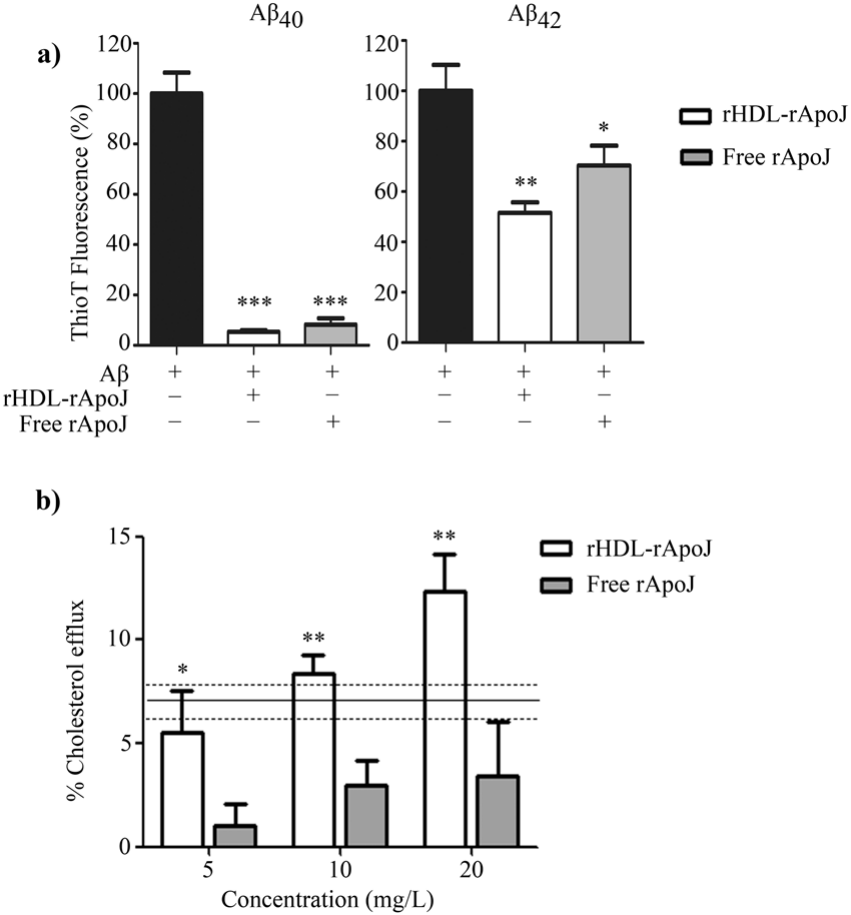
rHDL-rApoJ *in vitro* functionality. (**a**) Thioflavin T binding assay: ThT fluorescence intensity of Aβ_42_ and Aβ_40_ after 24 h at 37 °C with rApoJ or rHDL-rApoJ. Concentration ratio Aβ: free rApoJ/rHDL-rApoJ 1:0.01 (N = 4). One way ANOVA and Dunnett’s post hoc test: **p < 0.001, *p < 0.01 compared to control condition. **(b)** [^3^H] Cholesterol efflux removal from J774A.1 cells by rHDL-rApoJ and free rApoJ. The reference line indicates the mean ± SEM of the value obtained using ApoA-I (N = 3). t-test analysis: *p < 0.05, **p < 0.01 comparing free ApoJ vs. rHDL-rApoJ at each tested dose.

The functionality of rHDL-rApoJ nanodisc was studied through different strategies. First, we tested the chaperone-like activity exhibited by nanodiscs in comparison with free rApoJ measuring the ability preventing the fibrillization of Aβ_40_ and Aβ_42_ through the Thioflavin T (ThT) binding assay (Fig. 4a). We observed a high ability of both rApoJ and rHDL-rApoJ nanodiscs to avoid Aβ_40_ aggregation. In the case of Aβ_42_, both non-lipidated and lipidated rApoJ were effective preventing its fibrillization *in vitro*. No statistical differences regarding the effect of rHDL-rApoJ nanodiscs in comparison to free ApoJ were detected in any case. This result indicated that the ability of rApoJ to act as an Aβ chaperone was maintained despite its structural modification to form rHDL-rApoJ nanodiscs. On the other hand, the ability of rHDL-rApoJ nanodiscs to promote cholesterol ester efflux from cultured mouse macrophages was also analyzed (Fig. 4b). We determined that the RCT mediated by rHDL-rApoJ nanodiscs increased in a dose-dependent manner and was significantly higher compared to the cholesterol ester transport of free rApoJ at all the concentrations tested. As a reference value, 10 mg/L of rHDL-rApoJ removed the same percentage of esterified cholesterol as 10 mg/L of ApoA-I *in vitro*, highlighting the functionality of these particles in terms of cholesterol metabolism regulation. We confirmed that rHDL-rApoJ nanodiscs did not result cytotoxic *in vitro* (Supplementary Fig. S2).

### rHDL-rApoJ biodistribution in mice

**Figure 5:**
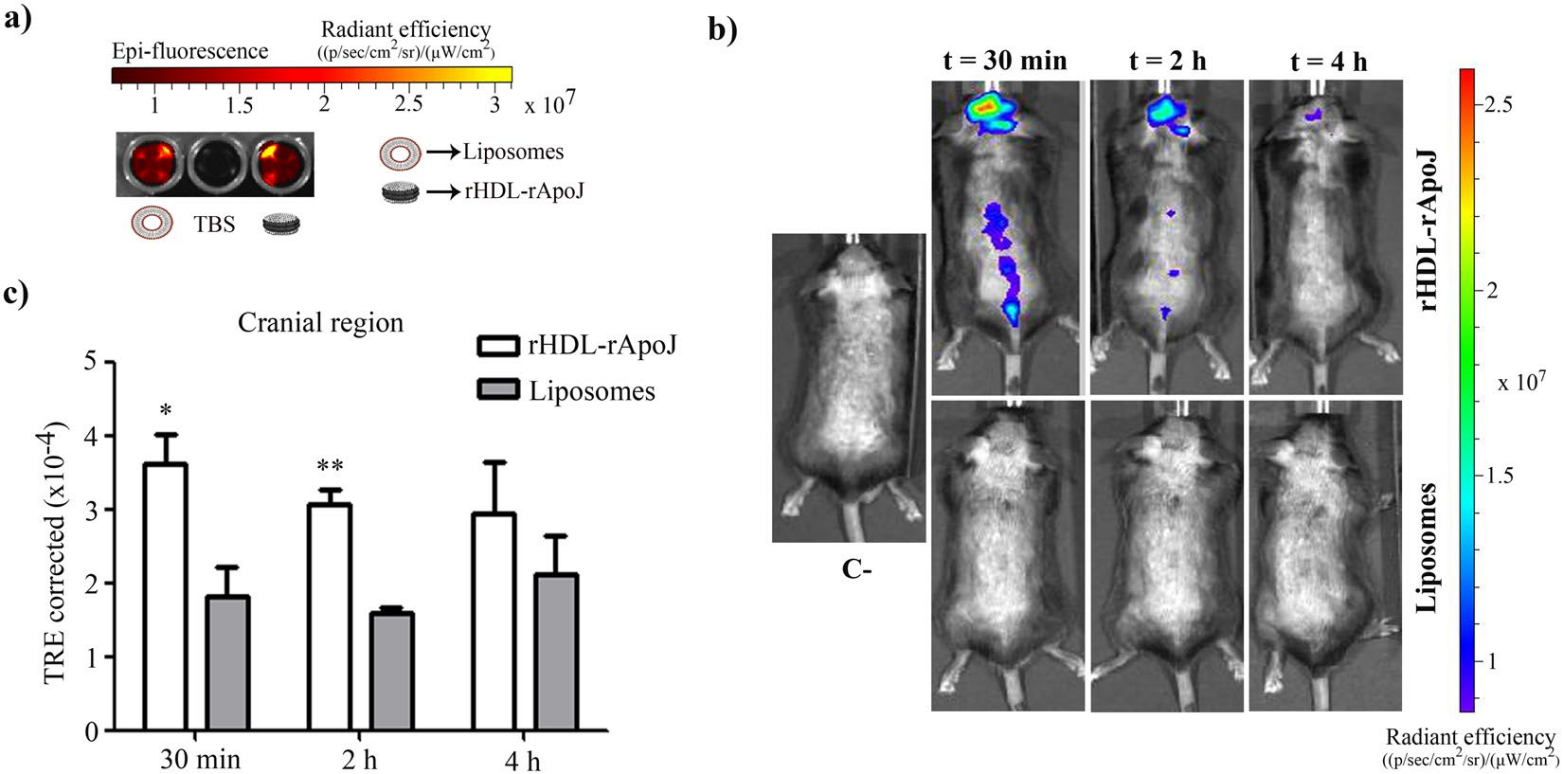
rHDL- rApoJ and liposome biodistribution in C57/BL6 mice. (**a**) IVIS Xenogen *in vitro* images of the epi-fluorescence of rHDL-rApoJ nanodisc and liposome preparations. **(b)** Representative *in vivo* IVIS Xenogen images of mice at 30 min, 2 h and 4 h after IV administration of labelled-rHDL-rApoJ or labelled-liposomes. C− = Non-treated control mouse. (**c**) Quantification of the fluorescent signal obtained in the cranial region after IV administration of labelled-rHDL-rApoJ nanodiscs or labelled-liposomes in 8 week-old C57/BL6 mice (N = 5–6/group). t-test analysis: *p < 0.05; **p < 0.01 signal from rHDL-rApoJ nanodiscs compared to liposome treatment at each time-point.

Once we had confirmed the functionality of the rHDL-rApoJ nanodiscs, we wondered whether these functional particles could reach the Central Nervous System (CNS). Therefore, we determined whether the rHDL-rApoJ nanodiscs could accumulate in the CNS after their peripheral administration in mice using the IVIS Xenogen system. For this purpose, as a control treatment, liposome particles with equal particle size and lipid composition as the rHDL-rApoJ nanoparticles were synthesized. We then compared the biodistribution of Alexa Fluor 750 (AF750) labelled rHDL-rApoJ nanodiscs and liposomes, after intravenous (IV) administration in young animals, by recording the fluorescent signal specifically in the cranial and spine regions at different time points. First, both preparations were analyzed *in vitro* using the IVIS Xenogen system, which demonstrated equal fluorescence in both samples (the fold-change increase relative to Tris Buffer Saline (TBS) of rHDL-rApoJ and liposomes was 15.1 and 14.8, respectively; Fig. 5a). After the infusion, rHDL-rApoJ showed a maximum record at 30 min in the cranial region, although the signal did not diminished significantly along the studied time-points. On the other hand, a higher signal in the cranial region was observed in mice treated with rHDL-rApoJ compared to liposome-treated mice at 30 min and 2 h after administration (Fig. 5b,c). In fact, after the background subtraction, no specific cranial accumulation was observed after the particle size-matched labelled-liposome administration (Fig. 5b), although the equal plasma detection and the bladder signal obtained after 4 h confirmed the correct infusion of both treatments (Supplementary Fig. S3). On the other hand, rHDL-rApoJ nanodiscs and liposomes were eliminated from circulation through renal clearance, as their fluorescent signal in the bladder increased over time. No differences in the fluorescent signal were observed in plasma 4 h after treatments, suggesting a similar elimination rate for rHDL-rApoJ nanodiscs and liposomes. No differences between groups were observed in the spine region either (Supplementary Fig. S3).

### rHDL-rApoJ retention in APP23 mice and wt mice

**Figure 6:**
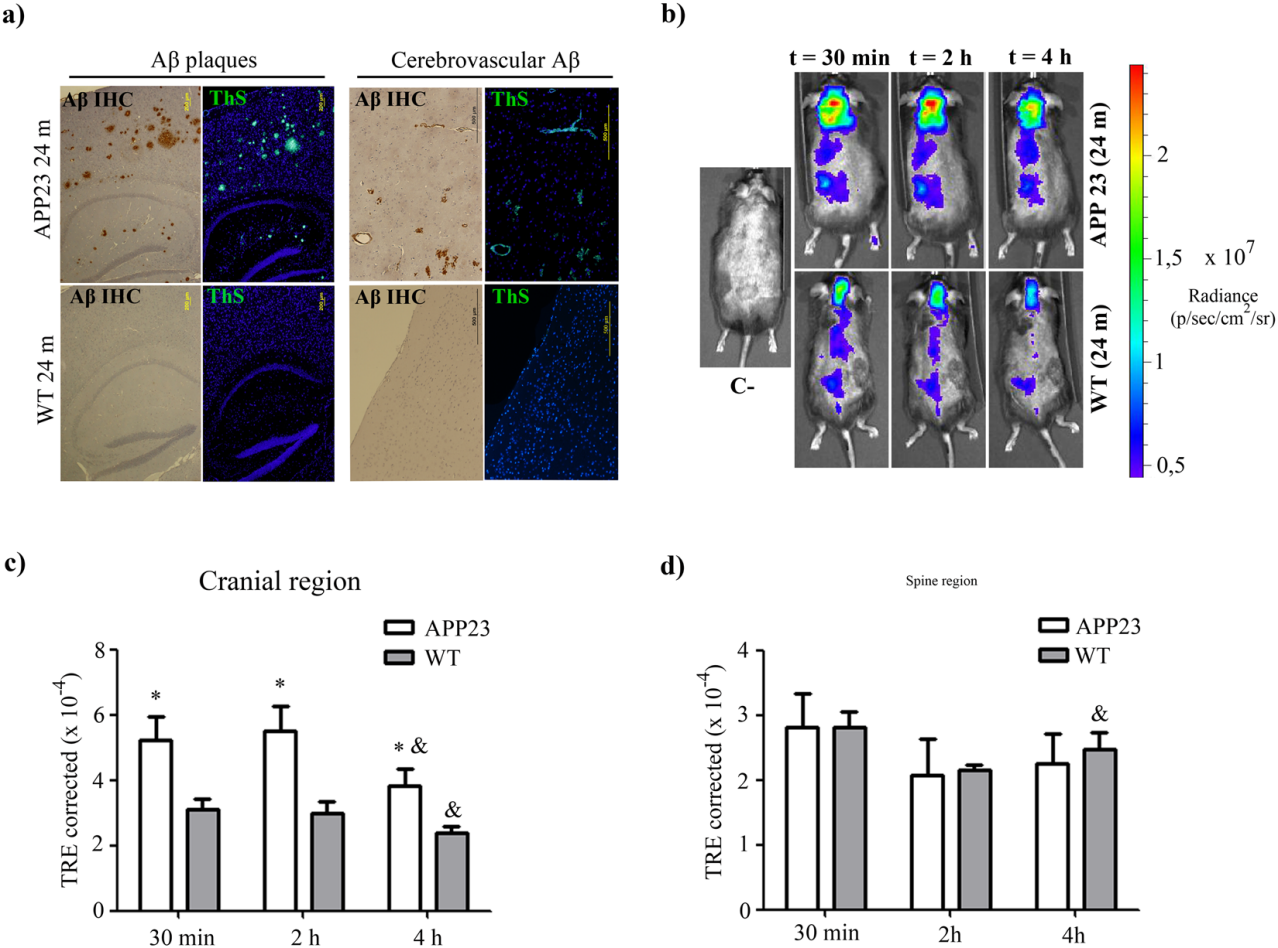
rHDL-rApoJ biodistribution in aged APP23 or wt mice. (**a)** Brain ThS staining and Aβ immunohistochemistry (IHC) from 24-month-old APP23 and wt mice showing the parenchymal and vascular accumulation of fibrillary Aβ. **(b)** Representative *in vivo* IVIS Xenogen images of mice at 30 min, 2 h and 4 h after IV administration of labelled-rHDL-rApoJ. C− = Non-treated control mouse. Quantification of the fluorescent signal obtained in the **(c)** cranial region and **(d)** spine region after IV administration of labelled-rHDL-rApoJ nanodiscs in 24-month-old APP23 and age-matched wt mice (N = 5–6/group). t-test analysis: *p < 0.05 comparison of the signal obtained in APP23 vs. wt mice at each time point. Paired t-test analysis: ^&^p < 0.05 comparison of t = 30 min and t = 4 h for each genotype.

Due to the high capacity of ApoJ to bind Aβ material^23,26^, we studied whether the distribution of peripherally administered fluorescent labelled-rHDL-rApoJ would vary in an *in vivo* model of cerebral β-amyloidosis. To accomplish this objective, we used 24-month-old APP23 mice, which is a well characterized transgenic model of AD^37^. In fact, Aβ immunohistochemistry and Thioflavin S (ThS) staining in brain slices from 24-month-old mice showed high accumulation of fibrillar Aβ material in the cortex, hippocampus and brain vessels of APP23 transgenic mice, whereas the corresponding wt littermates did not exhibit detectable Aβ deposition in the brain (Fig. 6a). In old mice, after the IV administration of labelled-rHDL-rApoJ and using the *in vivo* imaging system, we determined that the fluorescent signal diminished over time for both APP23 and wt animals in cranial, spine and liver regions (Fig. 6c,d). In turn, the fluorescent signal in the cranial region was higher in APP23 mice at all the time points analyzed in comparison to the signal obtained in the wt mice (Fig. 6b,c). These genotype-dependent differences were exclusive to the cranial region, as no differences were observed in the spine (Fig. 6c) and other body-regions studied, such as bladder, liver or in plasma 4 h after administration (Supplementary Fig. S4).

### rHDL-rApoJ cranial localization

**Figure 7:**
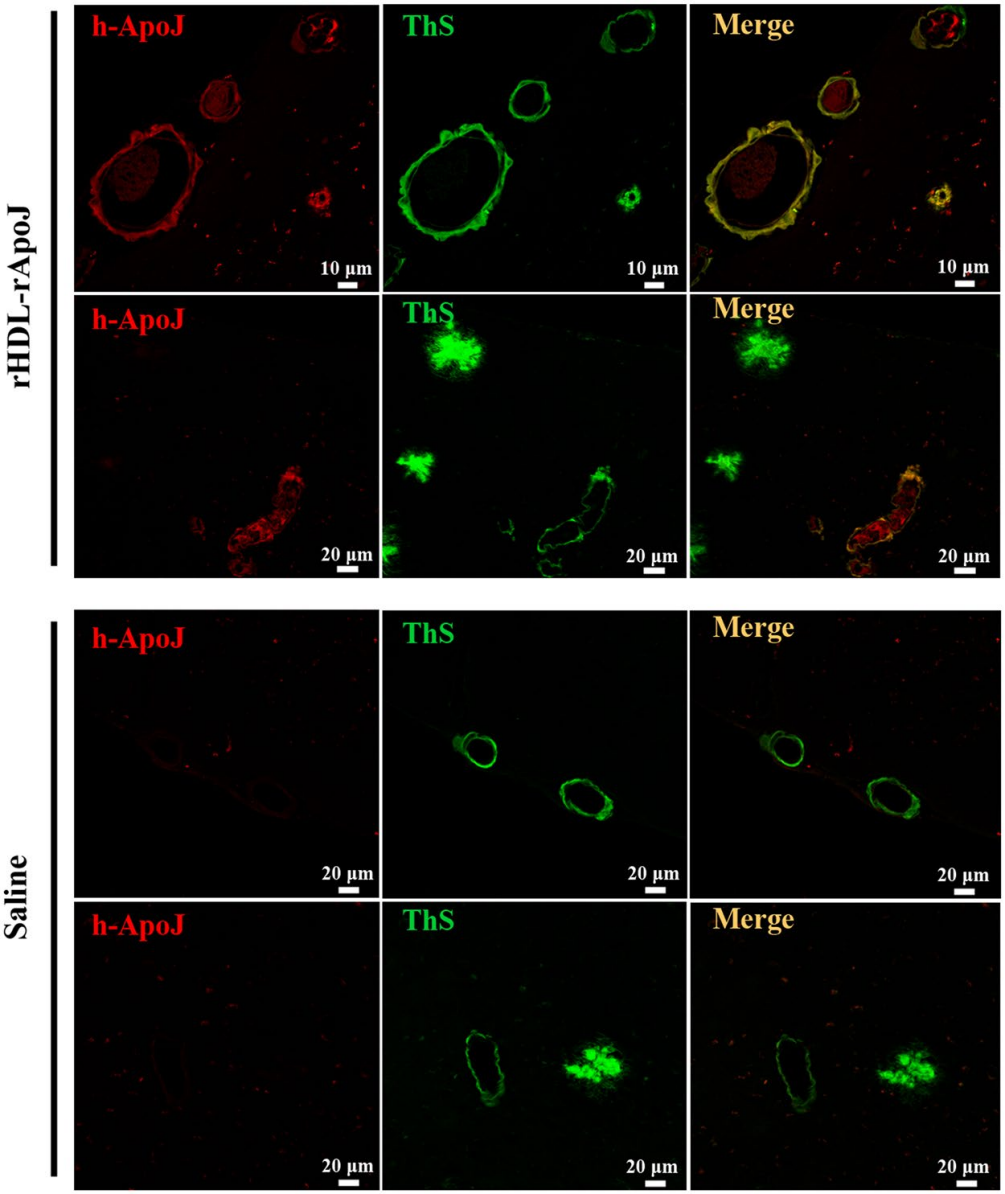
Localization of human rApoJ in brain after IV administration of rHDL-rApoJ nanodiscs in APP23 mice. Double labelling of human rApoJ (rApoJ in red) and fibrillary Aβ (ThS in green) in old APP23 animals treated with 1 mg/kg of rHDL-rApoJ nanodiscs or saline. Co-localization of rApoJ and fibrillary Aβ is observed in merged images (yellow).

In order to describe more insightfully the cranial accumulation of rHDL-rApoJ nanodiscs, we performed an immunofluorescence analysis to detect the presence of human rApoJ in brain sections of rHDL-rApoJ or saline-treated old APP23 mice. Under the studied conditions, we detected a specific co-localization of human ApoJ (from rHDL-rApoJ nanodiscs) with fibrillar Aβ (as shown by the positivity in ThS staining) in amyloid-affected cerebral vessels, whereas human ApoJ was not present in parenchymal amyloid plaques. In turn, human ApoJ was not detected in saline treated animals (Fig. 7).

## Discussion

Currently, there are no effective treatments to cure AD or to prevent the disease progression. Despite the fact that we are facing a multifactorial neurodegenerative disorder, recent evidence demonstrates that Aβ accumulation in AD is mainly explained by an impairment in its clearance from the brain^38^. Therefore, therapies based on molecules able to bind Aβ and promote its clearance are a plausible strategy to prevent the pathological hallmarks associated to AD. In this study, we present a new method to prepare and purify reconstituted HDL nanoparticles by assembling lipids with human recombinant ApoJ (rHDL-rApoJ). We consider that the treatment with rHDL-rApoJ may result in an increase in ApoJ circulating levels and this may be a promising strategy to treat pathologies associated with cerebral Aβ deposition because: (1) Some of the risk alleles of AD-associated SNPs in the CLU locus are associated with a decrease in ApoJ plasma levels^31^, whereas increased ApoJ circulating levels are associated with a higher prevalence of AD^27^, suggesting a protective role for high concentrations of plasma ApoJ in AD; (2) ApoJ is a natural Aβ chaperone, acting as the main Aβ escorting protein^34,35,39^; (3) Protective and anti-inflammatory properties have been attributed to ApoJ in experimental models, and particularly in cellular models challenged with Aβ^40,41^.

Back in the sixties, it was demonstrated that the mixture of the major constituents of HDLs (apolipoproteins, phospholipids and free cholesterol) under appropriate conditions resulted in the formation of discoidal shaped nanoparticles with comparable size and density to native Preβ-HDLs (density > 1.21 mg/ml, discoidal shape and without triglycerides and cholesterol esters in the core). These particles were called reconstituted HDL nanoparticles or nanodiscs (rHDL)^42,43^. Because our aim was to generate rHDL containing ApoJ, we first produced the recombinant protein from human cell cultures: this rApoJ was highly pure and conserved the heterodimeric nature and the propensity to form high molecular aggregates, as previously described for the human native ApoJ^44^. Next, we optimized the methodology to produce pure rHDL-rApoJ nanodiscs. To our knowledge, there is only one previous publication about rHDL nanodisc formulated with rApoJ^45^, although in that methodology a mixture of phospholipids and ApoJ purified from plasma were used. Complementary, the purpose of our study was to standardize a reproducible protocol to produce large amounts of rHDL-rApoJ for therapeutic purposes. Following the protocol here presented, we reproducibly obtained two populations with different hydrodynamic diameters of 30 ± 3 nm and 70 ± 1 nm as detected by DLS. N-PAGE analysis with posterior protein and lipid staining confirmed the presence of these two differently sized particles after the purification process with KBr density ultracentrifugation. The collection of different size population of rHDL synthesized with the cholate-dialysis method was previously observed for rHDL formulated with ApoA-I, where the diverse populations were attributed to the tertiary structure that the protein adopted^46^. Based in the DMPC:Chol:rApoJ molar ratio in purified rHDL-rApoJ sample (550:150:1) the small population is possibly composed by 1 molecule of rApoJ, whereas the bigger one could have at least 4 molecules of rApoJ, although this approximation needs to be further confirmed. In addition, TEM allowed the visualization of two-sized rHDL-rApoJ populations with a well dispersed discoidal shape. This nature is commonly observed in ApoA-I and ApoE containing rHDL, although in these cases nanoparticles usually present a more stacked distribution^47,48^.

Size distribution from TEM images confirmed the presence of two nanodiscs size populations whose mean diameters are centered on 24 ± 5 nm and 48 ± 2 nm respectively. The detection of smaller particles when size distribution was analyzed using TEM images in comparison to the ones obtained by DLS (hydrate state) is due to the dehydrated state of nanodiscs during TEM observation^49,50^. On the other hand, the discoidal morphology was confirmed using TEM, which allowed the edge-on and face-on visualization of the nanoparticles. Finally, nanodiscs were characterized using CD, which showed that the α-helical content of rApoJ increased 20.5% when the protein was assembled to form the rHDL structure. Indeed, an increase in the α-helical content was also described for ApoA-I nanodiscs^51^. This secondary structural change induced by the lipid-binding might have consequences regarding the functionality or stability of the protein. Regarding the bonding mechanism between lipids and rApoJ, it has been extensively described that apolipoprotein-lipid interaction in HDL particles is due to hydrophobic bonds between the hydrophobic chains of phospholipids and the anphipathic regions of apolipoproteins with α-helical structure^52,53^. The presence of 5 anphipathic α-helix in the structure of ApoJ^54^ support the idea of hydrophobic bonds between phospholipids and rApoJ. This binding has demonstrated to be stable to dilution and temperature effect^51^.

Therefore, the next step in our study was to analyze the *in vitro* functionality of the rApoJ-rHDL. First, we studied the chaperone*-*like activity exhibited by the nanodiscs in comparison to the free protein. We observed that both rHDL-rApoJ and free rApoJ were significantly effective in preventing Aβ_40_ and Aβ_42_ fibrillization. This chaperone-like ability is well established for ApoJ and it has been also observed for lipid-ApoJ complexes^39,45^. We also evaluated the regulation and transport of cholesterol esters, which is a relevant property of HDLs. In particular, we determined the cholesterol ester efflux ability of rHDL-rApoJ *in vitro* using cultured macrophages. rApoJ-nanodiscs showed a significantly higher activity in promoting the efflux of cholesterol esters than the free rApoJ, which was previously associated with cholesterol homeostasis^55^. We consider these results particularly relevant in relation to the treatment of β-amyloidosis neuropathologies. Cerebral atherosclerosis has been related with AD pathology^56,57^ and, while this issue has been a matter for discussion^58^, other authors have highlighted the relation existing between lipid metabolism and AD^6,14^. Therefore, we postulate that the administration of rHDL-rApoJ nanodiscs could promote the antiatherogenic properties of ApoJ and enhance its protective role in AD. In addition, studies performed in animal models of AD have shown that the genetic deletion of SR-BI^59^ or ABCA-1^60,61^, which are key mediators of the cellular cholesterol efflux pathway, induced an increase in fibrillar Aβ levels and cognitive worsening, suggesting that the modulation of cholesterol metabolism defines relevant features of the Aβ-associated pathology. Besides, some studies have highlighted the importance of ApoJ lipidation for its antiatherosclerotic properties^62^. Thus, although these results need to be confirmed *in vivo*, the possible enhancement of RCT by a pharmacological approach using rHDL-rApoJ may also bring promising results regarding brain Aβ accumulation levels. On the other hand, the successful brain delivery into CNS is one of the major challenges for the effective treatment of neurological disorders in genereal^63^ and AD in particular^64^. For this reason, we studied the biodistribution of rHDL-rApoJ nanodiscs after peripheral administration in mice in comparison with the distribution of size-matched liposome particles. We wondered whether the nanodisc structure obtained by the assembling of a lipid mixture with rApoJ was specifically able to be retained in the CNS. Administrating fluorescent-labelled nanodiscs or liposomes in young mice and using an imaging system *in vivo*, we observed higher cranial accumulation of rHDL-rApoJ nanodiscs 30 min and 2 h after IV infusion, although similar metabolism rates were observed for both treatments, according to plasma and bladder biodistribution. Focusing on the possible relevance of rHDL-rApoJ nanodiscs for the treatment of AD, we studied the biodistribution of rHDL-rApoJ nanodiscs in an experimental model of cerebral β-amyloidosis. In particular, we determined whether the elevated brain Aβ deposition shown in 24-month-old APP23 transgenic mice induced changes in the cerebral distribution pattern of the infused rHDL-rApoJ particles. Therefore, after IV administration, we followed the fluorescent-labelled particles *in vivo* in old transgenic mice and wt littermates, which allowed us to observe a consistent and specific higher accumulation of rHDL-rApoJ nanodiscs in the cranial region of APP23 animals at all the time points studied.

It has previously been described that ApoJ is able to cross the BBB through the megalin/LRP2 receptor^34,36^, therefore, one can speculate that the accumulation of cerebral Aβ also activates the *in vivo* recruitment of circulating ApoJ. Using fluorescent-labelling techniques in brain sections, we observed a specific accumulation of human ApoJ in Aβ-affected brain arterioles after an IV administration of rHDL-rApoJ nanodiscs. This evidence suggests that rHDL-rApoJ nanodiscs may be retained in brain vessels due to the binding of ApoJ to the specific receptor at a vascular level. The molecular mechanisms driving the specific entrapment of ApoJ in amyloid affected vessels has to be more deeply studied, although its chaperone function and the high affinity of ApoJ for Aβ seem to be crucial for the process. In fact, using an *in vitro* model of the BBB, our group showed that basolateral treatment with Aβ_40_ (corresponding to the brain side) enhanced the apical-to-basolateral influx of rApoJ^35^. Nevertheless, the present results did not allow us to conclude that rHDL-rApoJ nanoparticles intravenously administrated were able to penetrate the brain, since no human ApoJ was detected in parenchyma or bound to Aβ-plaques. Indeed, the absence of human ApoJ in parenchyma indirectly confirmed that the accumulation of fluorescent signal in rHDL-rApoJ treated-APP23 mice, shown in the IVIS analysis, was not likely to be due to a BBB leakage caused by cerebral β-amyloidosis. In fact, no parenchymal infiltration of trypan blue or horseradish peroxidase was observed in a previous study using very old APP23 mice^65^. Besides, it has recently been shown that BBB integrity remains intact in different AD mice models, as no differences were observed in the passage of different tracers and antibodies^66^. However, we cannot rule out that an alternative treatment design, as the infusion of a higher dose or an rHDL-rApoJ nanodiscs chronic administration, might bring out different results and a more extensive presence of the nanoparticles in parenchyma.

It has been demonstrated that ApoJ-Aβ complexes are more efficiently cleared than soluble Aβ through the megalin receptor^34,35^. Therefore, we hypothesize that treatment with rHDL-rApoJ nanodiscs may enhance the cerebral Aβ removal, as a higher megalin binding affinity of lipidated ApoJ has previously been demonstrated^45^. Taking these results together, the performance of future efficacy studies is essential to demonstrate whether a chronic peripheral treatment with rHDL-rApoJ nanodiscs reduces cerebral Aβ deposition and ameliorates the cognitive decline associated with the pathological features. On the other hand, the value of rHDL-rApoJ nanodiscs as a diagnostic tool to target vascular amyloid deposition merits further studies^67,68^.

Several arguments support the use of rApoJ-rHDL nanodiscs, rather than the recomobinant ApoJ, since the functionality of these nanoparticles can surpass the effect of the apolipoprotein. First, we have demonstrated that rApoJ-rHDL nanodiscs improved the cholesterol efflux transport compared to the free protein, which reinforces the use of HDL-based therapies in cerebral β-amyloidosis pathologies. Furthermore, lipid-based strategies have been shown to increase bioavailability and stability of proteins in plasma. Finally, the advancement of rHDL technology allows the functionalization of rHDL nanodiscs through a wide range of strategies^69^. Therefore, taking into account that rHDL-rApoJ nanoparticles are able to accumulate in brain vessels affected by Aβ deposition, rHDL-rApoJ may be theoretically functionalized with different therapeutic molecules to specifically target vascular Aβ.

In conclusion, we have designed a highly reproducible protocol for the formation and purification of large amounts of rHDL-rApoJ nanodiscs. We have demonstrated that the rHDL-rApoJ nanodiscs not only maintained the Aβ-chaperone functionality of rApoJ, but also improved *in vitro* cholesterol efflux abilities. We have also determined that rHDL-rApoJ nanodiscs accumulated in the cranial region of mice, especially in transgenic mice presenting a high cerebral Aβ load. Therefore, therapies based on rHDL-rApoJ nanodiscs may be considered for their potential use to treat neurological disorders associated to cerebral Aβ deposition.

## Methods

### Free recombinant ApoJ

The production and purification of rApoJ has been previously described by our group^31^. Briefly, rApoJ was produced in human cells (Human Embryonic Kidney 293T cells, HEK293T) and purified through Ni-affinity chromatography obtaining a purity >75%. The resulting recombinant protein was characterized by SDS-PAGE in both reductive and non-reductive conditions and by SEC. A detailed technical protocol for the production, purification and characterization of free rApoJ is supplied as Supplementary Information.

### Preparation of rHDL-rApoJ nanodiscs and liposomes

The nanodisc preparation protocol here presented is the result of an extensive optimization process. We tested the effect of different 1,2-dimyristoyl-*sn*-glycero-3-phosphocholine (DMPC, Avanti Polar Lipids, Alabaster, AL, USA) and rApoJ molar ratios (1000:1, 500:1, 200:1, 100:1 and 50:1) and different DMPC and free cholesterol (Chol, Sigma Aldrich, Saint Louis, MO, USA) molar ratios (5:1, 10:1 and 1:0). Besides, two lipid-solubilisation methods commonly used for the rHDL preparation were tested (spontaneous solubilisation method and cholate-dialysis method)^42,48,51^. Different nanodisc formation temperature (24 °C and 37 °C) and disc formation buffers (TBS or PBS) were tested. The selected conditions allowed high reproducibility of the rHDL-rApoJ nanodisc formation. First, a lipid mixture of DMPC and free Chol in chloroform solutions was prepared with a 5:1 DMPC:Chol molar ratio, the presence of free cholesterol in the lipid mixture enhanced the lipid-protein interaction^48^. The organic solvent was removed under vacuum and nitrogen to afford a dry lipid film, which was rehydrated with the disc formation buffer, TBS supplemented with 40 mM sodium deoxycholate (cholate, Sigma-Aldrich). This suspension was incubated at 37 °C for 30 min until a clear solution was obtained.

For rHDL-rApoJ nanodisc preparation, rApoJ and DMPC/Chol/cholate mixed micelles were mixed to afinal DMPC:Chol:rApoJ molar ratio of 550:110:1. Three incubation cycles of warming to 37 °C and freezing to 4 °C with vortexing were performed to promote rApoJ-DMPC interaction. After incubation, rHDL-rApoJ nanodisc self-assembly was initiated by cholate removal through extensive dialysis against a 1000-fold excess of TBS buffer at 4 °C for 48 h in 10,000 MW cut-off SnakeSkin dialysis tubing membranes (Thermo Fisher, Waltham, MA, USA) with two buffer changes (1 × 10^9^ overall dilution factor to ensure the correct nanodisc formation). Finally, unbound lipids were eliminated by sample centrifugation at 16,000 × g at 4 °C for 30 min. For the preparation of the liposome control without rApoJ, the liposomes were formed by cholate removal from the direct DMPC/Chol/cholate mixed micelles by extensive dialysis, as described.

The nanodiscs and liposomes were fluorescently labelled for specific experiments. To this end, Alexa Fluor 750 succinimidyl ester (AF750, ThermoFisher) and 1,2-dioleoyl-*sn*-glycero-3-phosphoethanolamine (DOPE-NH_2_, Avanti Polar Lipids) were conjugated as previously described^70^. Only conjugated AF750 was detected by thin-layer chromatography (Rf = 0.6), indicating that conjugation was complete. The fluorescently labelled AF750 rHDL-rApoJ nanodiscs and liposomes were prepared by incorporating AF750-DOPE into the lipid mixture (0.08 mM). The followings steps were not altered.

### Purification of rHDL-rApoJ nanodiscs with KBr density gradient ultracentrifugation

The rHDL-rApoJ nanodiscs were collected and separated from free rApoJ by potassium bromide (KBr; Sigma-Aldrich) density gradient ultracentrifugation^71^. After the ultracentrifugation step (100,000 × g, 24 h, 1250 mg/ml density of KBr), the sample was fractionated in 200 μl aliquots from top to bottom and quantification of lipids (DMPC and free cholesterol) and proteins of all fractions was performed using a Cobas c501/6000 autoanalyzer (Wako Chemicals GmbH, Neuss, Germany) and the BCA assay, respectively. Fractions with density <1,250 mg/ml containing the rHDL-rApoJ particles were dialysed together against TBS-Sucrose 2% (Sigma-Aldrich) to eliminate KBr from the solution. The lower density of rHDL-rApoJ nanodiscs in comparison with free rApoJ allowed the successful purification of rHDL-rApoJ nanodiscs and the total elimination of free rApoJ. The DMPC:Chol:rApoJ molar ratio in the purified rHDL-rApoJ sample was estimated to be 550:150:1 according to the lipid and protein quantification. Samples were stored at −80 °C.

### Characterization of rHDL-rApoJ nanodiscs

rHDL-rApoJ nanodiscs were characterized through different strategies. First, the colocalization of rApoJ and phospholipids, together with the molecular weight of nanodiscs were determined with N-PAGE in precast acrylamide gradient gels (BioRad, Hercules, California, USA) using fluorescent rHDL-rApoJ nanodiscs. Fluorescence of the lipids was detected using an ODYSSEY Imager (Li-Cor Biotechnology, Lincoln, NE, USA) followed by Coomassie staining for protein detection. Particle-size distributions of rHDL-rApoJ nanodiscs and liposomes were determined using a DLS analyzer combined with non-invasive backscatter technology (Malvern Zetasizer, Malvern Instruments, UK). The morphology of rHDL-rApoJ nanodiscs was determined by subjecting the sample to TEM with uranyl acetate negative staining. Far Ultraviolet (UV) CD spectrum was recorded to detect the conformational changes that occurred in rApoJ due to its lipidation. CD measurements (190–260 nm) were made of rApoJ and rHDL-rApoJ in TBS solutions in a JASCO815 spectropolarimeter. A detailed protocol of the techniques used for the rHDL-rApoJ characterization is supplied as Supplementary Information.

### ThT Binding assay

ThT binding methodology was used to compare the ability of free rApoJ and rHDL-rApoJ nanodiscs to avoid aggregation of Aβ_42_ and Aβ_40_ peptides in amyloid fibrils^72^. The Aβ_42_ and Aβ_40_ (Anaspec, Fremont, CA, USA) solutions were prepared from the lyophilized powder, resuspended in 1% NH_4_OH to a final concentration of 1 mM. The Aβ_40_ or Aβ_42_ and protein (rHDL-rApoJ or free rApoJ) concentration in the sample was 13 μM and 0.13μM respectively in a final volume of 150 μl in TBS solution (molar ratio Aβ: rApoJ 1:0.01). The fluorescence of each sample was measured in triplicate, mixing 40 µl of sample with 5 µl of ThT (Sigma-Aldrich; 0.1 mM) and 50 mM Tris buffer (pH = 8.5) until a final volume of 200 µl. Fluorescence was recorded in 96-well clear bottom plates after 300 s in a fluorometer (SynergyMx, Biotek, Winooski, Vermont, USA) (Ex/Em: 435/490).

### *In vitro* cholesterol ester efflux from J774A.1 cells

The *in vitro* ability of free rApoJ and rHDL-rApoJ nanodisc promoting cholesterol ester efflux was compared in mouse J774A.1 (ATCC ® TIB-67™) macrophage-like cells, as previously described^73^. Briefly, mouse J774A.1 macrophage-like cells (2 × 10^5^ cells/ml) were cultured in 6-well culture plates in Roswell Park Memorial Institute (RPMI) 1640 medium (Thermo Fisher Scientific) supplemented with 10 % Fetal Bovine Serum (FBS) (DDBiolab, Barcelona, Spain) and 1% penicillin/streptomycin (P/S), for 3 days at 37 °C. The cells were labelled with [1α,2α(n)-^3^H]cholesterol (GE Healthcare, 1 μCi per ml) for 48 h in RPMI 1640 medium, supplemented with 5% FBS, 1% P/S and acetylated Low Density Lipoproteins (LDL, 0.05 mg apoB/ml). The labelled cells were equilibrated overnight in RPMI 1640 medium containing 0.2% fatty acid-free Bovine Serum Albumin (BSA) and then incubated for 4 h at 37 °C with free rApoJ or rHDL-rApoJ nanodiscs (5, 10 and 20 mg/L). Human ApoA-I (10 mg/L; Sigma-Aldrich) was used as a positive control. Culture media were collected and centrifuged to discard detached cells. Then, 1 ml of 0.5 N NaOH was added to the wells and incubated overnight at 4 °C. Radioactivity was then measured in both culture media and NaOH-cell extracts, and the percentage of relative cholesterol ester efflux was calculated as the percentage of the [^3^H] counts released in the medium divided by the total [^3^H] counts (medium + cells).

### rHDL-rApoJ and free rApoJ citoxicity in hCMEC/D3 and SH-SY5Y cell cultures

The citotoxicity of 80 mg/L rHDL-rApoJ nanodiscs or free rApoJ was measured using the MTT (3-(4,5-dimethylthiazol-2-yl)-2,5-diphenyltetrazolium bromide; Sigma-Aldrich) reduction assay after 24 h in the cerebral human microvascular endothelial cell line (hCMEC/D3), provided by Dr. Couraud, Cochin Institute, France (Weksler *et al*., 2005) and in a neuroblastoma cell line (SH-SY5Y). The detailed methodology is extensively described previously^74^.

### Ethic statement for animal experimentation

All procedures were approved by the Vall D’Hebron Research Institute Ethics Committee for Animal Experimentation (protocol number 78/13) and conducted in compliance with Spanish legislation and in accordance with the relevant European Union Directives. All animals in the study were maintained in a climate-controlled environment on a 12-hour light/12-h dark cycle. Food and water were available *ad libitum*.

### rHDL-rApoJ nanodisc and liposome biodistribution *in vivo*

Fluorescently labelled rHDL-rApoJ nanodiscs and liposomes with equivalent size and lipid composition were used to monitor the biodistribution of administrated rHDL-rApoJ nanodiscs in mice. To ensure this equal lipid concentration and fluorescence, the DMPC concentration was quantified using the Cobas c501/6000 autoanalyser and adjusted to the rHDL-rApoJ DMPC concentration with TBS. Then, the comparable fluorescence was confirmed in 96-well black plates in the fluorometer (Ex/Em: 750/780). Besides, 50 μl of rHDL-rApoJ nanodiscs and liposomes were loaded in 96-well black plates and the epi-fluorescence was measured in an IVIS® Spectrum imaging system (PerkinElmer Inc., Waltham, MA, USA), located in the Preclinical Imaging Platform (PIP) of VHIR. For the *in vivo* experiments, eight-week old C57/BL6 mice (Janvier Labs, Le Genest-Saint-Isle, France) were used. The mice were shaved on their dorsal and ventral sides and anesthetized under spontaneous respiration with isoflurane (5%) in oxygen (2%). Next, 150 μl of fluorescent rHDL-rApoJ nanodiscs (100 μg/ml of rApoJ and 1.5 mM of DMPC) or fluorescent liposomes (1.5 mM of DMPC) were administrated IV via tail vein (N = 5–6/group). A non-treated animal was used for background extraction. Images of cranial, spine and bladder regions were acquired *in vivo* after IV administration of rHDL-rApoJ nanodiscs and liposomes at 30 min, 2 h and 4 h using the IVIS® Spectrum imaging system. EDTA-plasma was acquired through cardiac puncture at the 4 h time-point followed by euthanasia with decapitation under isoflurane anesthesia. Plasma fluorescence was quantified in 96-well black plates in the fluorometer. The results obtained from IVIS® Spectrum were analyzed using the Living Image 4.3.1 software (PerkinElmer Inc.). All data obtained by Xenogen IVIS® Spectrum experiments are expressed as Total Radiant Efficiency (TRE) considered a calibrated measurement of the photon emission from the subject and technically defined as fluorescence emission radiance per incident excitation intensity: photons/s/cm^2^/sr (steradian)/μW/cm^2^. TRE was corrected using the non-treated animal autofluorescence in each region, as well as the fluorescence of the infused sample measured in the fluorometer.

### rHDL-rApoJ nanodiscs biodistribution in old APP23 and wt mice

The biodistribution of administrated rHDL-rApoJ nanodiscs in old APP23 and wt littermate mice was studied. APP23 mice overexpress the APP protein containing the Swedish mutation (K670M/N671L) under the Thy1 neuronal promoter. Hemizygote B6, D2-TgN[Thy-APPSWE]-23-Tg mice (APP23) provided by Novartis Institutes for BioMedical Research, Novartis Pharma AG (Basel, Switzerland) were backcrossed twice with C57BL/6 mice (Janvier). The APP genotype was tested by Transnetyx (Cordova, TN, US) from tail*-*samples. For the biodistribution experiment using the Xenogen IVIS® Spectrum system, 24-month-old APP23 animals and age-matched wt mice were treated IV via tail vein (N = 5–6/group, 100 μg/ml rApoJ and 1.5mM DMPC) and images were obtained as previously described for young mice. A 24-month-old wt animal was used for background subtraction.

### Aβ Immunohistochemistry

For Aβ analysis in brain, 24-month-old APP23 and wt mice were anesthetized under isoflurane flow and euthanized, followed by transcardial perfusion with 25 ml of cold 0.1 M PBS, pH 7.4. Brains were rapidly removed, divided into hemispheres, and paraffin embedded. For the Aβ immunohistochemistry analysis, all samples were deparaffinized for 1 h at 65 °C and treated with 2% H_2_O_2_ and 10% methanol diluted in PBS for 15 min. The sections were incubated for 1 h in blocking solution (PBS, 0.2% triton-X, 10% FBS and 1.5 g/ml glycine) and, then, incubated overnight (ON) at 4 °C with an anti-human Aβ monoclonal antibody (Aβ4G8, Covance, Princeton, NY, USA) diluted 1:5000 in blocking solution. After that, slices were incubated with biotinylated α-mouse IgG (Vector Laboratories, Burlingame, CA, USA) diluted 1:200 in blocking solution for 1 h at RT and then for 1 h with streptavidin-HRP (Vector Laboratories) diluted 1:200 in blocking solution. Finally, diaminobenzidine (DAB) (Dako, Denmark) was applied until a brown end-product was visible and contrast staining was done with Harris hematoxylin solution (Sigma-Aldrich).

### ThS staining

Paraffin-embedded brain sagittal sections from 24-month-old APP23 and age-matched wt mice were used. All samples were deparaffinized for 1 h at 65 °C and immersed in 1% ThS dissolved in ethanol 75% during 30 s. The excess of ThS was drained and the slices were again immersed in 0.1% ThS in ethanol 75% for 1 min, then, slices were dehydrated and DAPI was used as contrast staining before mounting the preparations.

### Localization of rHDL-rApoJ nanodiscs in brain sections

To determine the cranial localization of rHDL-rApoJ nanodiscs after IV administration, 28 month old APP23 mice were treated with 1 mg/kg of rHDL-rApoJ nanodiscs via retroorbital injection. Animals were euthanized by decapitation 30 minutes after the administration and brains were rapidly removed, divided in two hemispheres and rapidly paraffin embedded. For immunofluorescence preparations, deparaffinization and blocking steps were done as previously described and anti-human ApoJ antibody (RD Bioscience, San Diego, CA, USA) diluted 1/100 in blocking solution was incubated ON at 4 °C. After that, slices were incubated with Alexa-Fluor594 labelled anti-mouse IgG (Invitrogen, Carlsbad, CA, USA) diluted 1/500 in blocking solution 1 h at RT. Finally, ThS staining was done as previously described and preparations were mounted.

### Statistical analysis

GraphPad Prism 5 was used for the statistical analysis. The significant differences were assessed using t-test, paired t-test and ANOVA with Dunnett’s multiple comparison post-hoc test, as appropriate. The variables are shown as mean ± SEM and a p-value < 0.05 was considered statistically significant.

### Data availability statement

The datasets generated during and/or analysed during the current study are available from the corresponding author on reasonable request.

## Electronic supplementary material

Supplementary Information
