# Supplementary material for: Characterization of ApoJ-reconstituted high-density lipoprotein (rHDL) nanodisc for the potential treatment of cerebral β-amyloidosis
Source: Sci Rep. 2017 Nov 7;7:14637. doi: 10.1038/s41598-017-15215-w (PMC5677083; doi:10.1038/s41598-017-15215-w)
Supplement: Supplementary file 1 — Supplementary Information [file 41598_2017_15215_MOESM1_ESM.pdf]

SUPPLEMENTARY INFORMATION

Characterization of ApoJ-reconstituted high-density lipoprotein (rHDL) nanodisc for the potential treatment of cerebral β-amyloidosis

Sofía Fernández-de-Retana[1], Mary Cano-Sarabia[2 *], Paula Marazuela[1], Jose Luis Sánchez-Quesada[3], Annabel Garcia-Leon[3], Alex Montañola[1], Joan Montaner[1], Daniel Maspoch[2,4], Mar Hernández-Guillamon[1 *]

[1] Neurovascular Research Laboratory, Vall d'Hebron Research Institute, Universitat Autònoma de Barcelona, Spain

[2] Catalan Institute of Nanoscience and Nanotechnology (ICN2), CSIC and the Barcelona Institute of Science and Technology, Campus UAB, Bellaterra, Barcelona, Spain

[3] Cardiovascular Biochemistry Group, Research Institute of the Hospital de Sant Pau (IIB Sant Pau), Barcelona, Spain

[4] Institució Catalana de Recerca i Estudis Avançats (ICREA), 08100 Barcelona, Spain

[*] **Corresponding Authors:**

Dr. Mar Hernández-Guillamon
Neurovascular Research Laboratory, Institut de Recerca, Pg. Vall d´Hebron, 119-129
Hospital Universitari Vall d´Hebron, 08035, Barcelona, Spain.
Phone N: +34934894029 Fax N: +34934894015
Mar.hernandez.guillamon@vhir.org / marguillamon@gmail.com

Dr. Mary Cano-Sarabia
Supramolecular NanoChemistry & Materials Group
Catalan Institute of Nanoscience and Nanotechnology (ICN2), CSIC and The Barcelona Institute of Science and Technology, Campus de la UAB, 08193, Barcelona, Spain
Phone N: +34937374637
Mary.cano@icn2.cat

SUPPLEMENTARY FIGURES

Supplementary Figure S1

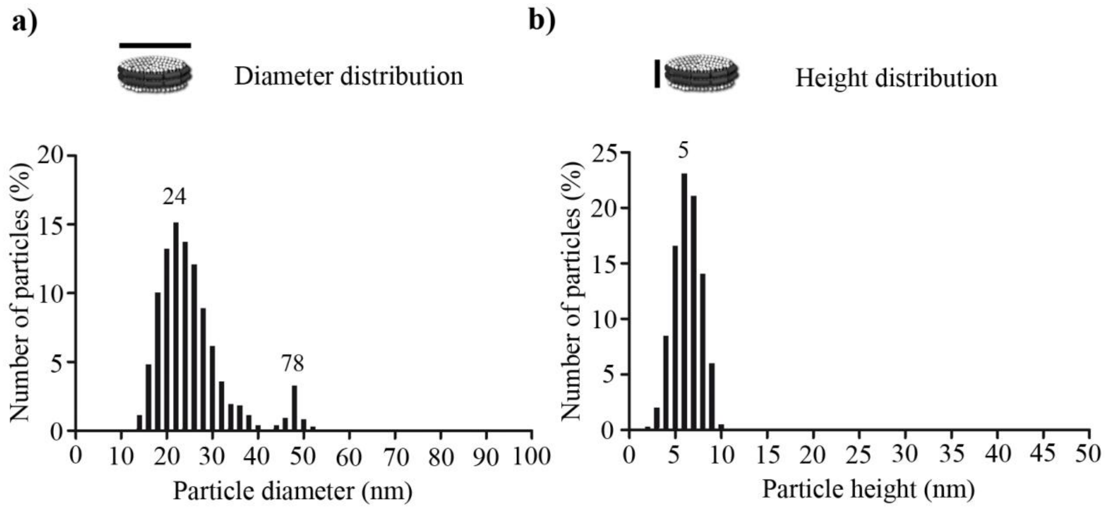

Supplementary Figure S1: Size distribution of purified rHDL-rApoJ nanodiscs at the ultrastructural level from the measurement of 1000 particles: a) Diameter distribution of purified rHDL-rApoJ nanodiscs. **b)** Height distribution of rHDL-rApoJ nanodiscs.

Supplementary Figure S2

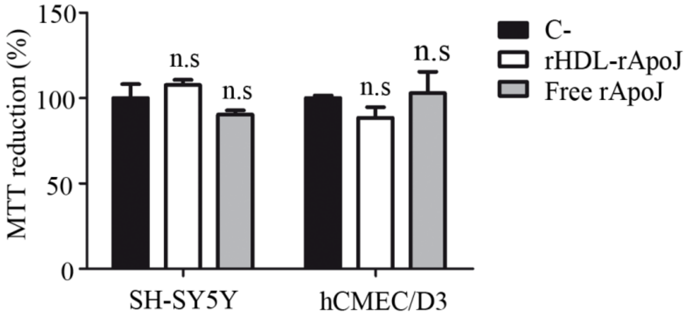

Supplementary Figure S2: Citotoxicity analysis of free rApoJ and rHDL-rApoJ nanodiscs. MTT reduction assay in cultured microvascular endothelial cells (hCMEC/D3) and neuroblastoma SH-SY5Y cells (as an in vitro model of neuronal function) after the treatment with rHDL-rApoJ nanodiscs or free rApoJ (80 mg/L) for 24 h. N=3; one way-ANOVA and Dunnett's post-hot test; n.s= non-significant.

Supplementary Figure S3

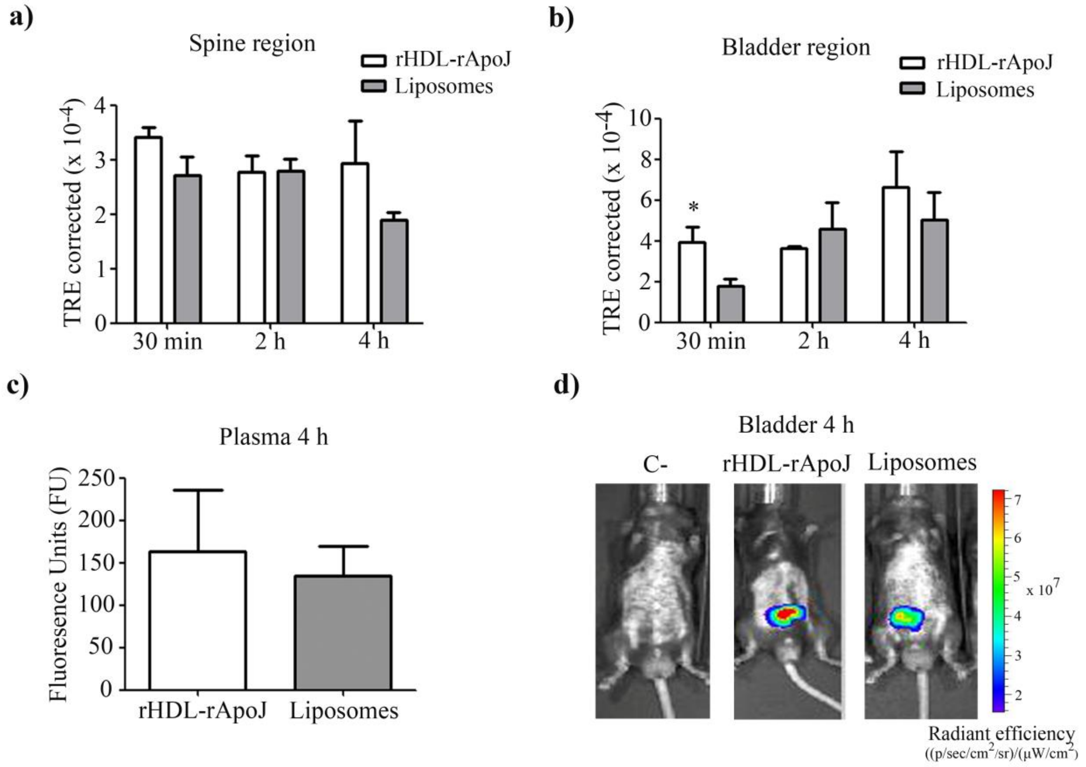

Supplementary Figure S3: Biodistribution of infused rHDL-rApoJ nanodiscs or liposomes in 8 week-old C57/BL6 mice. Quantification of the *in vivo* fluorescent signal obtained using the IVIS Xenogen system at 30 min, 2 h and 4 h after IV administration of labelled-rHDL-rApoJ or labelled-liposomes in **(A)** the spine and **(B)** the bladder of 8 week-old C57/BL6 mice, N=5-6/group. **(C)** Quantification of the fluorescent signal obtained in plasma from 8 week-old C57/BL6 mice treated with labelled-rHDL-rApoJ or labelled-liposomes determined at Ex/Em: 750/780, N=4-5/ group. **(D)** Representative IVIS Xenogen *in vivo* images of ventral mice sections at 4 h after IV administration of labelled-rHDL-rApoJ or labelled-liposomes. C- = Non-treated control mouse. t-test analysis; *$p < 0.05$ comparison of the signal obtained from mice treated with rHDL-rApoJ vs. liposomes at each time point.

Supplementary Figure S4

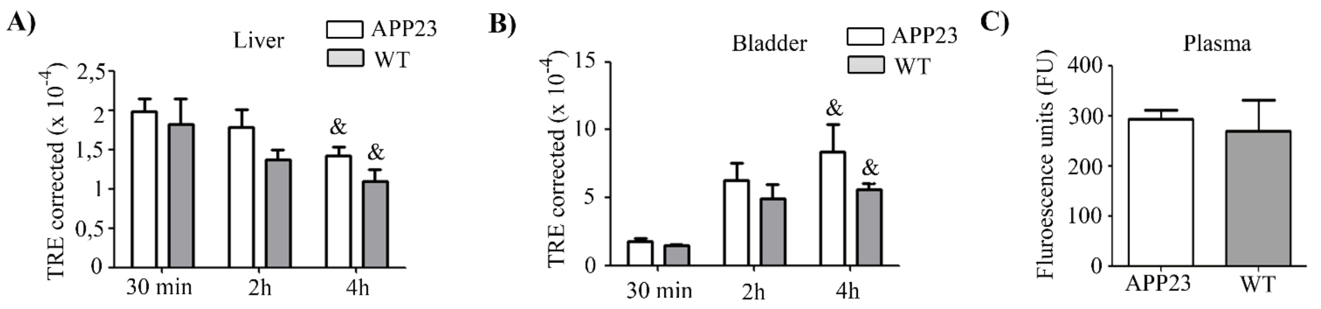

Supplementary Figure S4: Biodistribution of IV infused rHDL-rApoJ in aged APP23 and wt mice. Quantification of the *in vivo* fluorescent signal obtained using the IVIS Xenogen system at 30 min, 2 h and 4 h after IV administration of labelled-rHDL-rApoJ in **(A)** the liver and **(B)** the bladder of 24-month-old APP23 and wt mice, N=5-6/group. **(C)** Quantification of the fluorescent signal obtained in plasma from 24-month-old APP23 and wt mice 4 h after administration of labelled-rHDL-rApoJ determined at Ex/Em: 750/780. N=3/group. Paired t-test analysis; $^{\&}$ $p < 0.05$ comparison between t= 30 min and t = 4 h for each treatment.

SUPPLEMENTARY METHODS

Production, purification and characterization of free rApoJ

The production and purification of recombinant human ApoJ (rApoJ) has been previously described by our group[31]. Here below, we provide a detailed technical protocol for the production, purification and characterization of human free rApoJ:

Production and purification of free rApoJ: Human Embryonic Kidney 293T cells (HEK293T) were transfected with a pcDNA4.0™ vector containing human APOJ cDNA (Abgent, Clairemont, San Diego, USA). Stable transfected clones were cultured in HYPERFlask systems (Corning Inc., New York, USA), followed by supernatant recollection and purification. The protein was purified with Ni-affinity chromatography in a FPLC ÄKTA Purifier 100 System) with HiScreen Ni FF columns (GE Healthcare Bio-Sciences Corp., Piscataway, NJ, USA).Purified protein was dialyzed, lyophilized and reconstituted in Tris Buffer Saline (TBS, pH = 6.4) and its concentration was assessed using BCA assay (Thermo Fisher Scientific, Waltham, Massachusetts, USA). Aliquots were stored at -80ºC.

SDS-PAGE: The purity of rApoJ was determined using SDS-PAGE in 4-12% polyacrylamide gels under reducing (5% 2-mercaptoethanol and heating at 95 ºC for 5 min) and non-reducing conditions, followed by Coomassie SafeBlue staining (Nzytech, Lisbon, Portugal). The purity of rApoJ was > 75%, determined by analysis of the bands using the ImageJ software (rsweb.nih.gov). Human native ApoJ (nApoJ; Novus Biologicals, Littleton, CO, USA) was used as a positive control.

Size Exclusion Chromatography (SEC): The native state of free rApoJ protein was characterized by SEC in an ÄKTA Purifier 100 system. One-hundred µl of free rApoJ (4 mg/ml) was injected in a Superose 6 10/300 GL column (GE HealthCare Bioscience Corp.). Equilibration and elution were performed with Phosphate Buffered Saline (PBS) at 0.4 ml/min,

and the absorbance at 280 nm was monitored continuously obtaining a size dependent elution of different rApoJ aggregated species.

<u>Characterization of rHDL-rApoJ nanodiscs</u>

Once rHDL-rApoJ nanodisc where prepared and purified as described in the main text, resulting nanodiscs where characterized in terms of size, morphology and secondary structure of rApoJ. All the technical procedures used for the nanodiscs characterization are extensively described here below:

Native-PAGE: Purified rHDL-rApoJ particles were characterized by Native-PAGE in precast 4-20% acrylamide gradient gels (BioRad, Hercules, California, USA) using fluorescent rHDL-rApoJ nanodiscs. The Kit for High Molecular Marker 14,000-500,000 Non-Denaturing PAGE (Sigma-Aldrich) was used as molecular marker. A 25 µl sample aliquot was loaded and gels were run for 3 h at 140 V at 4ºC. The fluorescence of the lipids was detected using an ODYSSEY Imager (Li-Cor Biotechnology, Lincoln, NE, USA), recording the emission at 700 nm followed by Coomassie staining for protein detection.

Dynamic Light Scattering (DLS): Particle-size distributions of rHDL-rApoJ nanodiscs and liposomes were determined using a DLS analyzer combined with non-invasive backscatter technology (Malvern Zetasizer, Malvern Instruments, UK).. The mean of 3 different measurements was taken as the mean diameter.

Transmission Electron Microscopy (TEM): Isolated rHDL-rApoJ fractions were analysed by negative staining TEM. An 8 µl aliquot of rHDL-rApoJ nanodiscs was added to freshly glow-discharged carbon 300 Mesh copper grids (Ted Pella Inc. Redding, CA, USA) for 1 min. After blotting excess fluid, samples were stained with 8 µl of 5% uranyl acetate for 1 min. Samples were then examined in a JEM 1400 Transmission Electron Microscope (JEOL USA, Peabody, MA, USA). The diameter and height of 1000 discoidal particles was measured with ImageJ software.

Circular dichroism (CD): Far Ultraviolet (UV) CD spectrum was recorded to detect the conformational changes that occurred in rApoJ due to its lipidation. CD measurements (190-260 nm) were made of rApoJ and rHDL-rApoJ in TBS solutions (0.1 mg/ml) in a 1 mm path-length quartz cuvette in a JASCO-815 spectropolarimeter (JASCO Inc. Maryland, USA). The measurements were corrected by subtracting the spectra of a liposome solution in TBS (1 mM). The α-helical content was calculated from molar ellipticity at 222 nm ($[\theta_{222}]$) as previously described[1,2], using the Equation (1):

$$\alpha - helical\ content\ (\%) = \frac{-[\theta]_{222} + 3000}{39000} \times 100 \tag{1}$$

SUPPLEMENTARY REFERENCES

[1] Morrow, J. A. *et al*. Differences in stability among the human apolipoprotein E isoforms determined by the amino-terminal domain. *Biochemistry*. **39**, 11657-11666 (2000).

[2] WF. Zeno, W. F., Hilt, S., Risbud, S. H., Voss, J. C. & Longo, M. L. Spectroscopic characterization of structural changes in membrane scaffold protein entrapped within mesoporous silica gel monoliths. *ACS Appl Mater Interfaces*. **7**, 8640-8649 (2015).
